# Validation of a Classification Model Using Complete Blood Count to Predict Severe Human Adenovirus Lower Respiratory Tract Infections in Pediatric Cases

**DOI:** 10.3389/fped.2022.896606

**Published:** 2022-05-27

**Authors:** Huifeng Fan, Ying Cui, Xuehua Xu, Dongwei Zhang, Diyuan Yang, Li Huang, Tao Ding, Gen Lu

**Affiliations:** ^1^Department of Respiration, Guangzhou Women and Children’s Medical Center, Guangzhou Medical University, Guangzhou, China; ^2^Department of Immunology, Zhongshan School of Medicine, Sun Yat-sen University, Guangzhou, China; ^3^Pediatric Intensive Care Unit, Guangzhou Women and Children’s Medical Center, Guangzhou Medical University, Guangzhou, China; ^4^Key Laboratory of Tropical Disease Control, Ministry of Education, Sun Yat-sen University, Guangzhou, China; ^5^Provincial Engineering Technology Research Center for Biological Vector Control, Guangzhou, China

**Keywords:** human adenovirus, complete blood count, pediatric, severe, lower respiratory tract infection

## Abstract

**Background:**

Human adenovirus (HAdV) lower respiratory tract infections (LRTIs) are prone to severe cases and even cause death in children. Here, we aimed to develop a classification model to predict severity in pediatric patients with HAdV LRTIs using complete blood count (CBC).

**Methods:**

The CBC parameters from pediatric patients with a diagnosis of HAdV LRTIs from 2013 to 2019 were collected during the disease’s course. The data were analyzed as potential predictors for severe cases and were selected using a random forest model.

**Results:**

We enrolled 1,652 CBC specimens from 1,069 pediatric patients with HAdV LRTIs in the present study. Four hundred and seventy-four patients from 2017 to 2019 were used as the discovery cohort, and 470 patients from 2013 to 2016 were used as the validation cohort. The monocyte ratio (MONO%) was the most obvious difference between the mild and severe groups at onset, and could be used as a marker for the early accurate prediction of the severity [area under the subject operating characteristic curve (AUROC): 0.843]. Four risk factors [MONO%, hematocrit (HCT), red blood cell count (RBC), and platelet count (PLT)] were derived to construct a classification model of severe and mild cases using a random forest model (AUROC: 0.931 vs. 0.903).

**Conclusion:**

Monocyte ratio can be used as an individual predictor of severe cases in the early stages of HAdV LRTIs. The four risk factors model is a simple and accurate risk assessment tool that can predict severe cases in the early stages of HAdV LRTIs.

## Introduction

Human adenovirus (HAdV) plays a significant role in pediatric respiratory tract infections, accounting for 4–10% of pneumonia (1, 2). Life-threatening HAdV respiratory tract infections have previously been described in immunocompromised patients (3, 4). Although most immunocompetent children are mild and indistinguishable from other viral causes, lower respiratory tract infections (LRTIs) caused by HAdV can be severe or even fatal, and are associated with the highest risk of long-term respiratory sequelae (3, 5). Some studies have also shown that HAdV is closely related to severe pneumonia in children, accounting for 20–33.3% of severe pneumonia cases (6–8). HAdV infections are the leading cause of death in children with severe pneumonia, with a fatality rate of up to 12% and a risk of up to 14–60% of developing long-term respiratory complications such as post-infectious bronchiolitis obliterans (PIBO) and bronchiectasis (9–11). During epidemics of HAdV, some of the children with severe HAdV LRTIs require transfer to pediatric intensive care units (PICUs) because of disease progression and/or combined with other complications (5, 12). Therefore, early diagnosis and prompt intervention are very important for reducing the incidence of respiratory sequelae and protecting patients’ lives.

The earlier symptoms of pediatric patients with HAdV LRTIs are fever and cough (2, 13), and it is difficult to discern severe cases. Currently, the diagnosis of severe HAdV LRTIs mainly depends on the clinical symptoms that appear in the late stage of the disease (14, 15). Biochemical markers such as procalcitonin (PCT), C-reactive protein (CRP), lactate dehydrogenase (LDH), and tumor necrosis factor (TNF) in combination with chest radiography findings help to identify patients at risk as well as to determine appropriate treatment methods (16, 17). Studies have demonstrated that proadrenomedullin (Pro-ADM) and interleukin-1β (IL-1β) are thought to have potential for the evaluation of community-acquired pneumonia (CAP) in children (16). However, their accessibility for the prediction of severity in the early stages of HAdV LRTIs remains to be established for children. So far, there are no readily accessible biomarkers that can be used to assess disease severity and simplify the diagnosis process at the early stage of HAdV LRTIs.

Routine blood tests are a group of tests that evaluate cells that circulate in the blood. This is an important and convenient indicator of body health. Complete blood count (CBC), including red blood cells, white blood cells, and platelets, can detect systemic inflammation and infection status (18). However, patterns, deviations, and relations between blood parameters are too complex for clinicians to make accurate disease diagnoses. In contrast, machine learning algorithms can easily process high-dimensional data, and they can detect and exploit interactions between these numerous features (19, 20), which have important applications in disease detection, diagnosis, and personalized medicine by modeling blood parameters (20–22).

To date, few studies have focused on the early diagnostic value of CBC parameters for severe HAdV LRTIs. In the present study, we used the random forest model based on an ensemble-learning algorithm to select the most important CBC features and to establish a clinical prediction model in order to predict severity in children with HAdV LRTIs at an early stage.

## Materials and Methods

### Study Population, Data Collection and Ethics

We conducted a chart review to identify 1,258 patients aged ≥1 month to 18 years old with a diagnosis of HAdV LRTIs at Guangzhou Women and Children’s Medical Center, a tertiary pediatric hospital in southern China. The study period extended from January 1, 2013 to December 31, 2019. Clinical information and follow-up information of children with HAdV LRTIs were collected during the study period. Data were analyzed from March 1, 2020 to June 30, 2021.

This study followed the Strengthening the Reporting of Observational Studies in Epidemiology (STROBE) reporting guidelines. The study protocol was conducted in accordance with the Declaration of Helsinki and approved by the Ethics Committee of Guangzhou Women and Children’s Medical Center of Guangzhou Medical University (202049600). All patients provided written informed consent for the use of their clinical and laboratory data from their medical reports.

### Definitions

Lower respiratory tract infections were diagnosed definitively by the presence of both clinical (i.e., febrile respiratory illness and respiratory tract signs) and radiological evidence (23). All radiographic reports were obtained by trained pediatric radiologists. The chest radiograph images were re-checked by the investigators to confirm the diagnosis of LRTIs.

Human adenovirus infection was identified by positive multiplex polymerase chain reaction (PCR) for HAdV from nasopharyngeal swabs, sputum, and bronchial alveolar lavage fluid (BALF) (1).

The exclusion criteria for the study were as follows: severe concomitant diseases (chronic pulmonary diseases such as asthma, severe cardiovascular disease, neoplasia, epilepsy, severe neurological diseases, and kidney or liver disease), immunosuppressive states (chemotherapy, post-transplantation such as bone marrow transplantation, and use of immunosuppressive medications before disease onset, such as glucocorticoids), and immunodeficiency diseases (primary immunodeficiency and acquired immunodeficiency syndrome).

Severe or mild HAdV LRTIs were classified based on their clinical features. The diagnosis of severe cases was obtained when the following criteria were fulfilled (14): (1) Major criteria: invasive mechanical ventilation; fluid refractory shock; acute need for non-invasive positive pressure ventilation; hypoxemia requiring a fraction of inspired oxygen (FiO_2_) greater than the inspired concentration or flow feasible in the general care area. (2) Minor criteria: respiratory rate greater than the World Health Organization classification for age; apnea; increased work of breathing (e.g., retractions, dyspnea, nasal flaring, or grunting); partial pressure of arterial O_2_(PaO_2_)/FiO_2_ ratio <250; multilobar infiltrates; pediatric early warning score >6; altered mental status; hypotension; presence of effusion; and unexplained metabolic acidosis. Clinicians should consider providing care in an intensive care unit or a unit with continuous cardiorespiratory monitoring for children with ≥1 major or ≥2 minor criteria.

For early diagnosis, we selected CBC specimens within 7 days of onset to screen for predictors and develop an available model. The time, measured in days since the onset of fever (temperature ≥37.5°C) and/or cough, was the initial symptom of HAdV LRTIs.

### Blood Values and Cell Ratios

Complete blood count samples of pediatric patients were collected from their peripheral blood. The red blood cell count (RBC), hemoglobin (HGB), hematocrit (HCT), mean corpuscular volume (MCV), mean corpuscular hemoglobin (MCH), mean corpuscular hemoglobin concentration (MCHC), red cell distribution width-CV (RDW-CV), red cell distribution width-SD (RDW-SD), white blood cell count (WBC), neutrophil ratio (NEUT%), lymphocyte ratio (LYMPH%), eosinophil ratio (EO%), basophil ratio (BASO%), monocyte ratio (MONO%), band form neutrophilic granulocytes (NEUT Band cells), platelet count (PLT), mean platelet volume (MPV), plateletcrit (PCT), and platelet distribution width (PDW) were determined using the CBC test. We also collected metadata such as gender, age, clinical manifestations, time intervals between onset of symptoms and CBC test.

### Statistical Analysis

Clinical data were analyzed using the GraphPad software (Prism 8.0). Due to the skewed distribution of the clinical data, medians with interquartile ranges (IQRs) were used to express the summarized data. The non-parametric Mann–Whitney *U* test was used for the two-group analysis of continuous variables. Categorical variables were assessed using Fisher’s exact test.

We performed principal component analysis (PCA) (24) to reduce the dimension of the data by recombining the original variables into new independent variables and retaining the information of the old variables as much as possible. Following the PCA analysis, we selected the two most important principal components of the variables. For continuous metadata, such as hospital stays of inpatients or time intervals between onset of symptoms and CBC test, Pearson’s correlation analysis was used to analyze the correlation between the first principal component (PC1) and continuous metadata. The first principal component (PC1) axis can well represent data characteristics by showing the largest variation in the data as possible. Statistical significance was set at *p* < 0.05.

The CBC tests of the patients in this study were sampled at multiple time points. For each CBC parameter, we performed time-series analysis for severe and mild cases. To identify if specific CBC parameters were differentially expressed in severe and mild cases, we used MetaLonDA (25). And 999 permutations were performed to ensure meaningful *p*-values. To select only the significant associations, we chose a threshold of 0.05 for the *p*-values following the FDR adjustment. The scatters were smoothed using the Loess method by default in the time series curve.

In this study, data were analyzed using R 3.6 software^1^ (26) and the data visualization was based on the ggplot2 package (27). Pmsampsize package of R software (version 3.6.1) was utilized to calculate the sample size required for developing a clinical prediction model.

### Random Forest Classification Model Development and Evaluation

The study used Random forest (RF) algorithm which is one of the most frequently used AI algorithms for classification purposes in clinical research studies. It combines the output of multiple decision trees rising from the training subset to solve for regression or classification problems. It was characterized by anti-overfitting and antinoise abilities. RF was used as a classification method in this study. We used proximity of random forest trained on the observed values of a data matrix to predict the missing values. The data set was defined as the discovery and validation cohort according to the year of sampling. Here, we specified a random seed number of 1234, and the number of trees was set to 1000 for the discovery cohort. The RF model adopted the bootstrap sampling method in which N participants were randomly selected from the dataset and included in the training subset. During model training, the simulations were repeated 10 times using 10-fold cross-validation. The final determination of the RF model classification was based on the majority votes. The importance ranking of features that is MeanDecreaseGini was evaluated in the classification task using the decision trees of the RF model. It is calculated the effect of variables such as CBC parameters in our study on the heterogeneity of observations at each node of the classification tree to compare the importance of variables. Mean decrease in the Gini index was used to rank various features based on their importance. When the higher the Gini index value, the more important is the value given by the variable. The validation cohort was then classified based on the classification model. To test the predicted effectiveness of our model and verify its representativeness of our discovery cohort, we used the area under the receiver operating characteristic curve (AUROC).

In this study, the missing values in the data set were imputed by the miss Forest package in R (28). We performed classification analysis using the random forest method, and the ROC curves were obtained using the pROC package (29).

Here, we retained the most important predictors in the discovery cohort using the RF model. To generate simple classification criterions to aid diagnosis, we selected the important CBC parameters on severity of HAdV LRTIs patients and a conditional inference tree was implemented by the conditional inference tree (CTREE) function of partykit package (30).

## Results

### Cohort Description

**FIGURE 1:**
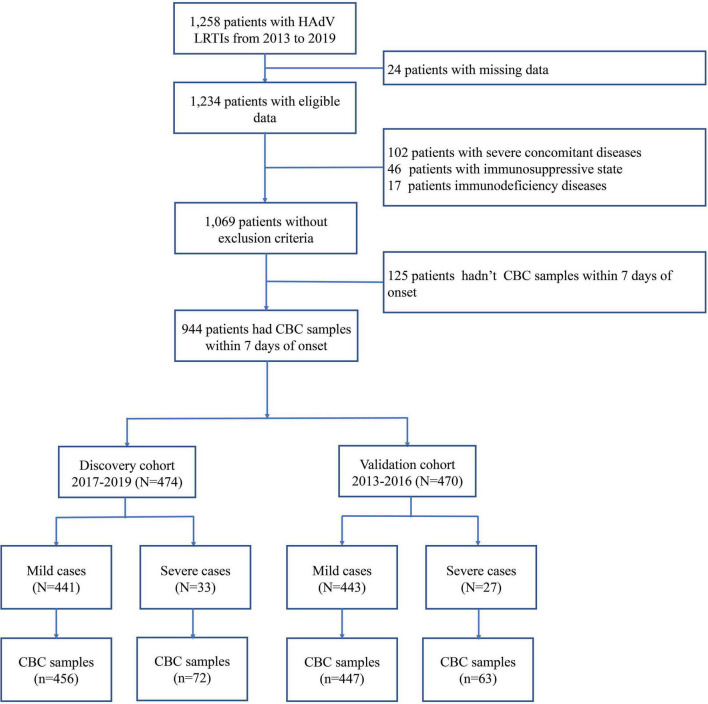
Flow diagram.

In the present study, 1,069 pediatric patients with newly diagnosed HAdV LRTIs were included, following the inclusion and exclusion criteria (Figure 1). The study included 1,652 CBC specimens from 1,069 pediatric patients, including 1,329 specimens from mild cases and 323 specimens from severe cases. There were 645 boys (60.34%) and 424 girls (39.66%), with a median age of 35 months (IQR: 19–48 months). The median interval between the onset of symptoms and diagnosis was 4 days (IQR: 3–6 days). There were 1,000 children with mild HAdV LRTIs and 69 children with severe HAdV LRTIs. The main clinical symptoms of HAdV LRTIs were fever (1,047/1,069, 97.94%), cough (986/1,069, 92.23%), and wheezing (251/1,069, 23.48%). The total incidence of sequelae was 4.96% (53/1,069) in all patients, and the total mortality was 2.06% (22/1,069).

As shown in Figure 1, 944 patients were included within 7 days of onset to establish an early prediction model. Based on different years of diagnosis, 474 patients, which were derived from 2017 to 2019, were used as the discovery cohort, and 470 patients from 2013 to 2016 were used as the validation cohort. There were no statistically significant differences in demographic characteristics, symptoms, complications, treatment, and outcomes between the two cohorts (Table 1).

**TABLE 1:** Clinical features of patients in two cohorts.

Items	Discovery cohort (*N* = 474)	Validation cohort (*N* = 470)	*p* value
**Demographic**			
Age (months), M (IQR)	36 (19–52.75)	36 (20–48)	0.6175
Male, No. (%)	290 (61.18)	273 (58.09)	0.3532
Severe cases, No. (%)	33 (6.96)	27 (5.74)	0.5053
**Symptoms**			
Fever, No. (%)	461 (97.26)	452 (96.17)	0.3673
Cough, No. (%)	435 (91.77)	441 (93.83)	0.2574
Wheeze, No. (%)	121 (25.53)	108 (22.98)	0.3635
Tachypnea, No. (%)	78 (16.46)	65 (13.83)	0.2762
Increased work of breathing (e.g., retractions, dyspnea, nasal flaring, and grunting), No. (%)	33 (6.96)	27 (5.74)	0.5053
**Severe complications**			
ARDS, No. (%)	18 (3.80)	24 (5.11)	0.3477
Fluid refractory shock, No. (%)	8 (1.69)	2 (0.43)	0.1075
MODS, No. (%)	11 (2.32)	9 (1.91)	0.8219
**Treatment**			
High-flow oxygen therapy, No. (%)	33 (6.96)	27 (5.74)	0.5053
Non-invasive positive pressure ventilation, No. (%)	15 (3.16)	10 (2.13)	0.4182
Mechanical ventilation, No. (%)	30 (6.33)	24 (5.11)	0.4840
CBP, No. (%)	4 (0.84)	3 (0.64)	0.9999
ECMO, No. (%)	5 (1.05)	3 (0.63)	0.7254
**Outcomes**			
Sequelae, No. (%)	24 (5.06)	19 (4.04)	0.5330
In-hospital mortality, No. (%)	8 (1.69)	7 (1.49)	0.9999

*ARDS, Acute Respiratory Distress Syndrome; MODS, Multiple Organ Dysfunction Syndrome; CBP, Continuous blood purification; ECMO, Extracorporeal membrane oxygenation.*

### Dimension Reduction Analysis of Complete Blood Count Parameters

**FIGURE 2:**
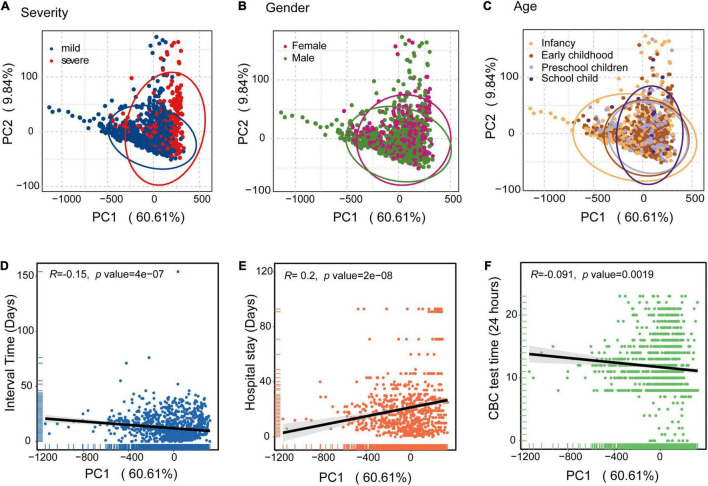
Principal component analysis (PCA) of the blood biochemical values applied to the clinical information of HAdV LRTIs. **(A)** Scatter plot for the PCA analysis of blood biochemical values by severity. **(B)** Gender groups of the patients. **(C)** Age cohorts of the patients. **(D)** Correlation between PC1 and the interval times (Days) between onset of symptoms and CBC test data of the patients. **(E)** PC1 values and hospital stays (Days) of the patients. **(F)** PC1 values and CBC testing times (24 h) of day for the patients. The black line represents the fitting line.

To avoid the influence of age, gender, and CBC test time differences, we analyzed the CBC parameters based on principal component analysis (PCA). PCA plots were based on all CBC measurements for the 474 children in the discovery cohort. The variance proportions of principal component 1 (PC1) and principal component 2 (PC2) were 60.61 and 9.84%, respectively. PCA analysis of severity showed that children with severe disease were largely clustered separately from those with mild disease (Figure 2A). Severe individuals diverged from mild individuals along the PC1 axis, indicating that PC1 can explain an important component of the variance that separates mild and severe individuals. This suggests that severe cases of HAdV LRTIs significantly alter multiple blood parameters. However, the differences of gender and age were not significant (Figures 2B,C). In addition, we identified interval times that had negative loading and hospital stays with positive loading on PC1, which may indicate that there is a significant association between mild/severe cases and hospital stays/intervals between symptom onset and CBC test (Figures 2D,E). However, there was no significant association between the CBC test time and the PC1 axis (Figure 2F).

### Dynamic Trajectories of Complete Blood Count Parameters

**FIGURE 3:**
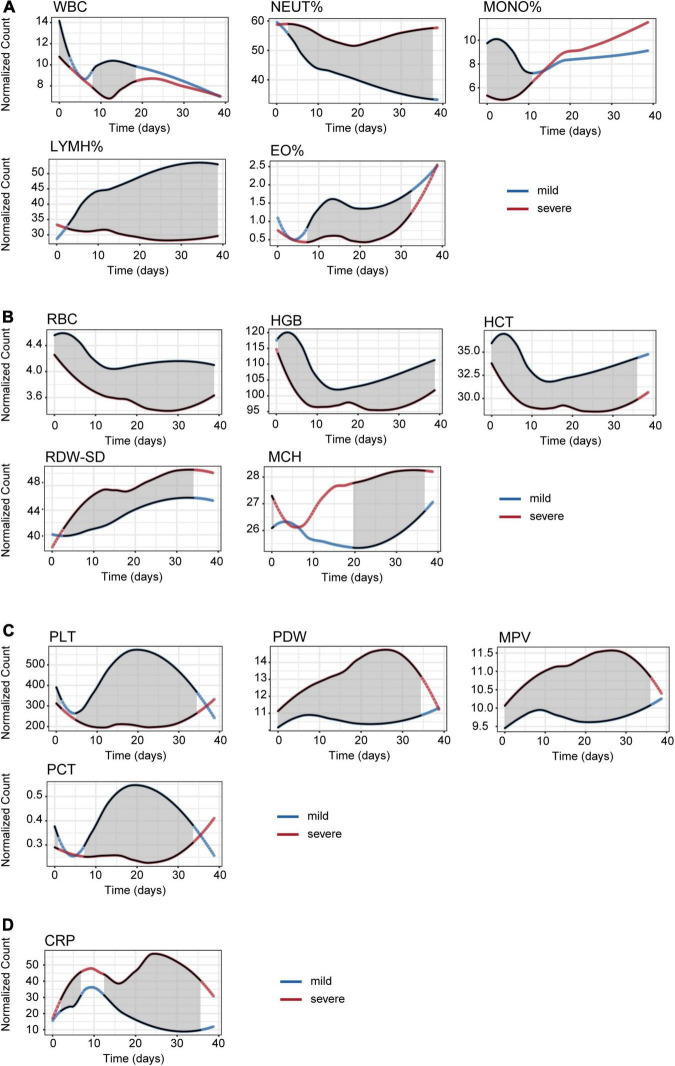
Time series analysis of the CBC indices for mild and severe patients. **(A)** Leukon related biochemical values. **(B)** Erythron related biochemical values. **(C)** Megakaryocytic related biochemical values. **(D)** C-reactive protein values.

Figure 3 illustrates the linear fitting curve for dynamic trajectories of 15 important CBC parameters from onset to day 40 of the disease course grouped by disease severity, and the other parameters are shown in Figure 1. The shaded areas indicate significant differences between the two groups. We found some of the same types of blood cells had similar dynamic trajectories for mild and severe cases. For leukon series such as NEUT% and LYMPH%, the difference between the mild and severe groups was not significant at the early stage. However, the difference between them significantly increased with the disease progression (Figure 3A). RBC, HGB, and HCT counts which belongs to erythron series, were significantly declined early on admission and persisted low throughout the 40-day period of onset compared to mild cases (Figure 3B). As to EO% and megakaryocytic series such as PLT, PCT, PDW, and MPV, differences between the two groups were not significant in the early and late stages, but significant differences were observed between 5 and 35 days after onset (Figure 3C). For the CRP, MCH, RDW-SD, PDW, and MPV values, an upward trend was observed in the severe cases during the 40 days (Figure 3D). In contrast, the levels of MONO%, LYMPH%, EO%, RBC, HGB, HCT, PLT, and PCT decreased with disease severity. In particular, there was a significant difference between severe and mild cases in terms of MONO% in the early stage, which indicates that MONO% has a significant difference and indicative effect in the early stage of HAdV LRTIs. Therefore, we infer it is an important indicator of disease severity at the early stage.

### Performance of Monocyte Ratio in the Discovery and Validation Cohorts

**FIGURE 4:**
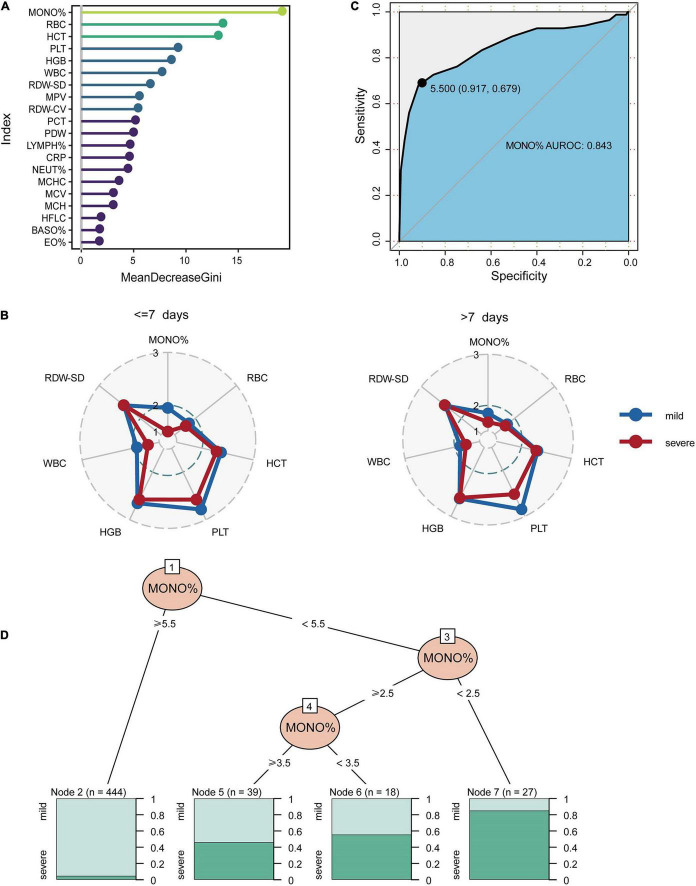
The classification performance of MONO% at the early stages of HAdV LRTIs. **(A)** Feature importance ranking of the discovery cohort in the RF model. **(B)** Concentrations which is represented by the deciles of biomarkers of the top six important biomarkers within 7 days or more than 7 days after onset. **(C)** Area under the receiver operating characteristic curves of MONO% for the discovery cohort. **(D)** Conditional inference tree (CTREE) displaying MONO% identified as significant split nodes using the non-parametric regression method. Numbers along the branches indicate split values of variance-stabilized blood indices. The terminal nodes show the proportion of samples originating from patients with different degrees of severity.

The minimum sample size was suggested 452 pediatric patients by the pmsampsize, and we enrolled 474 pediatric patients (528 specimens) from 2017 to 2019 as the discovery cohort according to sample size requirements. Random forest model was used to classify patients with mild and severe symptoms using various blood indicators. The most important feature selected by the MeanDecreaseGini index was MONO% for the discovery cohort (Figure 4A). The concentrations of the top six important biomarkers within 7 days or more than 7 days after onset are shown. As demonstrated, early presenters within 7 days had a significantly lower MONO% than those in the late stage (Figure 4B), and AUROC for MONO% was 0.843. The cut-off for the patients in which there was optimal discrimination between mild and severe individuals was 5.5. This score provided the best trade-off between sensitivity (91.7%) and specificity (67.9%) (Figure 4C). MONO% was considered the best single predictor, and it was used in a further decision tree analysis using partykit. The best threshold of MONO% in the root node was <5.5 versus ≥5.5 for the first step. For the node that included patients with MONO% values of 5.5 or higher, 98 percent of the cases (*n* = 444) were mild. In people with MONO% less than 2.5, 88% (*n* = 27) were severe HAdV LRTIs cases. Patients were more likely to develop severe cases when MONO% value was lower than 2.5 (Figure 4D).

### Predictor Selection and Module Development Performance of the Classification Model in the Discovery and Validation Cohorts

**FIGURE 5:**
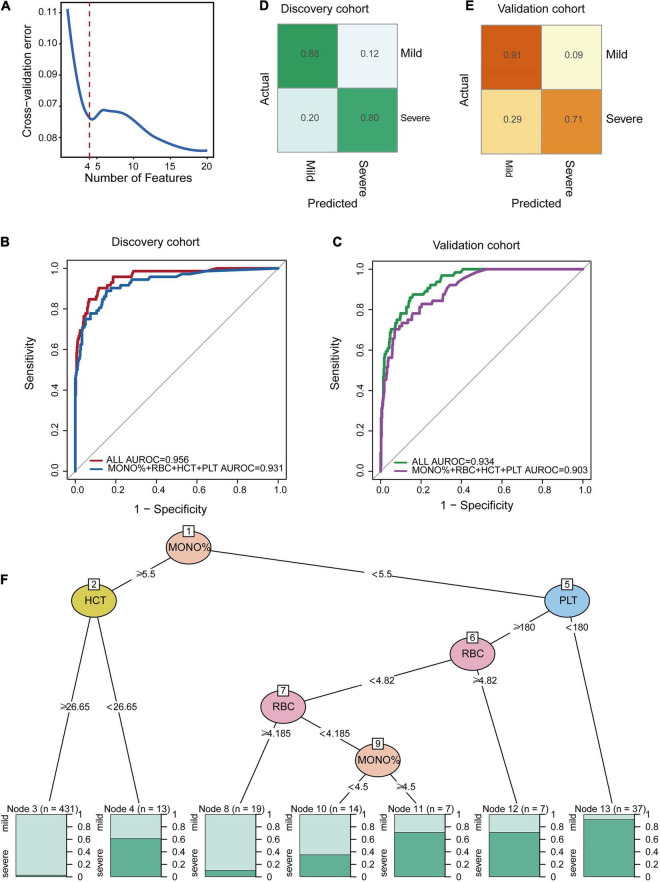
Random forest classification model at the early stage of HAdV LRTIs. **(A)** Cross-validation error of the discovery cohort in the RF model. **(B)** Area under the receiver operating characteristic curves of the four features of the discovery cohort. **(C)** Area under the receiver operating characteristic curves of the four features of the validation cohort. **(D)** Confusion matrix of the discovery cohort. **(E)** Confusion matrix of the validation cohort. **(F)** Conditional inference tree (CTREE) displaying the four blood indices identified as significant split nodes using the non-parametric regression method. Numbers along the branches indicate split values of variance-stabilized blood indices. The terminal nodes show the proportion of samples originating from patients with different degrees of severity.

As it is difficult for a single index to consider sensitivity and specificity, this study combined multiple indicators to build a classification model. We found that the classification performance of the comprehensive index was significantly higher than that of the single index. Based on the relatively low repeated cross-validation error and fewer selected features, we identified four indicators as the optimal number of features (Figure 5A). According to the feature importance in Figure 4A, we found the classification model constructed by combining MONO%, RBC, HCT, and PLT values had the best effect. The ROC curves and the AUC values of the stratified 10-fold cross-validation are presented in Figure 5B, in which the AUROC of all CBC parameters and for the four important indexes of the discovery cohort were 0.956 and 0.931, respectively. The normalized confusion matrix corresponding to fold 10 is shown in Figure 5D. We validated the accuracy and specificity of the classification model for patients in the validation cohort. In the validation cohort, the classification effect of the classifier was also excellent. The AUROC of all CBC parameters was 0.934, and the AUROC of the four important indexes was 0.903 for the validation cohort and confusion matrix were also shown (Figures 5C,E).

To provide an interpretable overview of this RF predictive model, we also constructed a decision tree model, as shown in Figure 5F, which approximates the implementation of the RF model. The partykit method identified the MONO% from the four important CBC metrics as the best single discriminator between mild and severe patients. The best predictor in the root node was the MONO%, using a <5.5 versus ≥5.5 threshold for the first step. The node with MONO% ≥ 5.5 and HCT ≥ 26.62 provided diagnostic value for 98 percent of mild patients (*n* = 431). For the node with MONO% < 5.5 and PLT < 180, 94 percent of the cases (*n* = 37) were severe. For the node with MONO% < 5.5, PLT ≥ 180, and RBC ≥4.185, 90 percent of the cases (*n* = 19) were mild. Therefore, this model can be used as a simple decision-making aid for clinicians who use blood indexes to evaluate the severity of HAdV LRTIs.

## Discussion

Human adenoviruses (HAdV) are non-enveloped, double-stranded DNA viruses that can cause respiratory tract diseases in children (1). Previous studies have shown fatal cases of severe HAdV pneumonia caused by particular serotypes, even in immunocompetent patients (15, 31, 32). Therefore, it is vital to predict the development or progression of severe HAdV LRTIs at an early period, and the high sensitivity and specificity of early predictors of severe cases have not been systematically studied in immunocompetent pediatric patients. Analysis of complete blood count (CBC) parameters’ changes caused by HAdV LRTIs in children mainly focused on the classification of severity and the changes in CBC parameters at different sampling times in order to seek the CBC parameters of targeted diagnosis, treatment, and related to the prognosis of HAdV LRTIs in children.

From the time series analysis of CBC parameters, the monocyte ratio (MONO%) was the most obvious difference between the mild and severe groups at the disease onset. Monocytes, bone marrow-derived blood-resident phagocytes, are recruited under pathological conditions (such as viral infections) to the affected tissue to defend the organism against invading pathogens and to aid in the efficient resolution of inflammation (33, 34). Recent studies have suggested that circulating monocytes and tissue macrophages participate in all stages of coronavirus disease 2019 (COVID-19) (35, 36). They contribute to comorbidities predisposing one to clinical infection, virus resistance and dissemination, and to host factors that determine disease severity, recovery, and sequelae (35). The level of circulating monocytes depends on the circadian release from the bone marrow, adhesion to endothelial surfaces, and tissue entry. Blood from COVID-19 hospitalized patients has shown a decrease in the proportion of monocytes, consistent with the release of immature, more frequently replicating Ki67+ monocytes from the bone marrow during emergency hematopoies (37). Similar to our study, MONO% levels in severe cases were significantly lower than in mild cases in the early stage and may serve as a key pathway in HAdV infections; thus, their reduction may be partly responsible for disease progression, especially in the early stages of HAdV infections. Therefore, MONO% might be used as a single marker for the early and accurate prediction of the disease’s severity. The results of the current study revealed a certain finding with a large number of subjects, and the AUROC of MONO% in predicting severe cases was high enough for clinical application (AUROC: 0.843). In addition, we developed a machine-learning model. While the proportion of monocytes was lower, the possibility of severe cases was higher. Pediatric patients with HAdV LRTIs were likely to develop a severe form when MONO% value was lower than 2.5. This cut-off value was sufficient to separate those at high-risk and low-risk of developing severe forms of the disease at the early stage, even before the appearance of clinical manifestations. Decreased MONO% values could be an additional warning sign for clinicians to pay more attention to a patient’s potential of developing severe HAdV LRTIs at an early stage.

Subsequently, we applied the random forest (RF) algorithm to build and validate a predictive model for early HAdV LRTIs. The model included four features (MONO%, RBC, HCT, and PLT) based on the Gini coefficient and model error rate. The diagnostic performance of this established model was excellent in both the discovery cohort from 2017 to 2019 and the validation cohort from 2013 to 2016. Since the grouping of mild and severe cases was imbalanced, we employed the RF method that was based on a bagging type ensemble learning algorithm (38). RF divides the training data into multiple subsamples while ensuring that each subsample is fully balanced. The RF ensemble learning method outperformed support vector machine (SVM), bagging, and boosting for unbalanced data (39). Therefore, the model may serve as an accurate and reliable tool for quickly quantifying the risk of severity across different clinical cohorts. Because the CBC test is one of the most commonly available and low-cost tests, the classification model can be readily determined and implemented as a very simple and economical tool to prioritize patients quickly, particularly for physicians with diverse backgrounds and specialties. The machine-learning model showed good performance in discriminating the risk of severity in all groups. If a patient’s predicted risk for severity is low, the doctor may choose to monitor at peripheral or district hospitals, while a high-risk estimate might support the use of more aggressive interventions or the need for an early transfer to a tertiary center or admission to an intensive care unit. The classification model can help physicians allocate limited resources to the areas with less advanced healthcare systems during HAdV pandemics.

Although our study developed and validated a simple and applicable model based on a large cohort of HAdV LRTIs, several limitations should be noted when interpreting the results. First, this simple CBC model could be a convenient tool for stratifying patients at risk in clinics; however, it may have limited ability to explain all variances across patient populations. Second, the patient cohort was recruited from hospitalized patients from only one province in China. Whether this model can be generalized to patients with different genetic and geographic backgrounds requires further external validation. Third, the types of HAdV were not analyzed in the CBC model because the types in most of the HAdV LRTI cases were not examined. Even if there was a lack of HAdV types, the CBC model still distinguished severe cases. This indicates that the CBC model has wider applications.

## Conclusion

In this study, we found that MONO% can be used as an individual predictor of severe cases in the early stage of HAdV LRTIs. More importantly, we developed and validated a readily applicable risk assessment tool to dynamically estimate the risk of severity in children with HAdV LRTIs during their disease course by using CBC parameters. As the CBC test is the most commonly available test, the classification model may assist pediatric clinicians in providing early diagnoses and prompting interventions for children with severe HAdV LRTIs.

## Data Availability Statement

The original contributions presented in the study are included in the article/supplementary material, further inquiries can be directed to the corresponding authors.

## Ethics Statement

The studies involving human participants were reviewed and approved by the Ethics Committee of Guangzhou Women and Children’s Medical Center of Guangzhou Medical University. Written informed consent to participate in this study was provided by the participants’ legal guardian/next of kin. Written informed consent was obtained from the individual(s), and minor(s)’ legal guardian/next of kin, for the publication of any potentially identifiable images or data included in this article.

## Author Contributions

HF and YC performed the research and wrote the manuscript. DY, DZ, LH, and XX collected the data. TD and YC analyzed the data. GL and TD performed the study design and critical revision. All authors read and agreed to the published version of the manuscript.

## Conflict of Interest

The authors declare that the research was conducted in the absence of any commercial or financial relationships that could be construed as a potential conflict of interest.

## Publisher’s Note

All claims expressed in this article are solely those of the authors and do not necessarily represent those of their affiliated organizations, or those of the publisher, the editors and the reviewers. Any product that may be evaluated in this article, or claim that may be made by its manufacturer, is not guaranteed or endorsed by the publisher.

## Footnotes

1
https://www.r-project.org/
